# Scale-Free Functional Brain Networks Exhibit Increased Connectivity, Are More Integrated and Less Segregated in Patients with Parkinson’s Disease following Dopaminergic Treatment

**DOI:** 10.3390/fractalfract6120737

**Published:** 2022-12-13

**Authors:** Orestis Stylianou, Zalan Kaposzta, Akos Czoch, Leon Stefanovski, Andriy Yabluchanskiy, Frigyes Samuel Racz, Petra Ritter, Andras Eke, Peter Mukli

**Affiliations:** 1 Department of Physiology, Semmelweis University, 1094 Budapest, Hungary; 2 Institute of Translational Medicine, Semmelweis University, 1094 Budapest, Hungary; 3 Berlin Institute of Health at Charité, University Hospital Berlin, Charitéplatz 1, 10117 Berlin, Germany; 4 Department of Neurology with Experimental Neurology, Charité-University Hospital Berlin, Corporate Member of Freie Universität Berlin and Humboldt Universität zu Berlin, Charitéplatz 1, 10117 Berlin, Germany; 5 Vascular Cognitive Impairment and Neurodegeneration Program, Oklahoma Center for Geroscience and Healthy Brain Aging, Department of Biochemistry and Molecular Biology, University of Oklahoma Health Sciences Center, 975 NE 10th Street, BRC, Oklahoma City, OK 73104, USA; 6 Department of Neurology, Dell Medical School, University of Texas at Austin, Austin, TX 78712, USA; 7 Bernstein Focus State Dependencies of Learning and Bernstein Center for Computational Neuroscience Berlin, 10115 Berlin, Germany; 8 Einstein Center for Neuroscience Berlin, Charitéplatz 1, 10117 Berlin, Germany; 9 Einstein Center Digital Future, Wilhelmstraße 67, 10117 Berlin, Germany; 10 Department of Radiology and Biomedical Imaging, Yale University School of Medicine, 300 Cedar Street, New Haven, CT 06520, USA

**Keywords:** multifractal, functional connectivity, brain networks, electroencephalogram, Parkinson’s disease

## Abstract

Dopaminergic treatment (DT), the standard therapy for Parkinson’s disease (PD), alters the dynamics of functional brain networks at specific time scales. Here, we explore the scale-free functional connectivity (FC) in the PD population and how it is affected by DT. We analyzed the electroencephalogram of: (i) 15 PD patients during DT (ON) and after DT washout (OFF) and (ii) 16 healthy control individuals (HC). We estimated FC using bivariate focus-based multifractal analysis, which evaluated the long-term memory (H(2)) and multifractal strength $\left(\Delta H_{15}\right)$ of the connections. Subsequent analysis yielded network metrics (node degree, clustering coefficient and path length) based on FC estimated by H(2) or $\Delta H_{15}$. Cognitive performance was assessed by the Mini Mental State Examination (MMSE) and the North American Adult Reading Test (NAART). The node degrees of the $\Delta H_{15}$ networks were significantly higher in ON, compared to OFF and HC, while clustering coefficient and path length significantly decreased. No alterations were observed in the H(2) networks. Significant positive correlations were also found between the metrics of H(2) networks and NAART scores in the HC group. These results demonstrate that DT alters the multifractal coupled dynamics in the brain, warranting the investigation of scale-free FC in clinical and pharmacological studies.

## 1. Introduction

Parkinson’s disease (PD) is the second most common neurodegenerative disease, after Alzheimer’s disease, with a prevalence of more than 1.5% in people above the age of 65 [1]. A gradual loss of dopaminergic neurons in the substantia nigra constitutes the characteristic pathological change of PD. Dopamine depletion leads to impaired extrapyramidal motor function, which manifests as tremor, bradykinesia, freezing of gait and muscular rigidity. These motor symptoms are the pathognomonic features of PD. However, non-motor symptoms-such as autonomic dysfunction, affective symptoms, olfactory loss or sleep disturbance-develop at earlier stages and are sometimes misidentified [2], having a more profound impact on the central nervous system. Dopaminergic treatment (DT) has been the mainstay of motor-specific therapy, which can improve motor symptoms fast and efficiently. However, the unstable pharmacokinetics of DT leads to over- or under-stimulation depending on dopamine’s plasma levels. In addition, the stimulation of the nigrostriatal pathway coincides with the activation of other dopamine pathways that are not involved in motor control, namely the mesolimbic, mesocortical and tuberoinfundibular pathway. Psychosis, hallucinations and addiction are well-known side effects of DT, manifested through those accessory pathways. The potentially widespread effects of DT have led to large-scale investigations of functional connectivity (FC) in PD.

Neuroimaging studies of resting-state FC allowed the identification of PD- and DT-related changes in brain networks [3], while functional magnetic resonance imaging (fMRI) allows for the reconstructing of FC of the brain with high spatial resolution, based on the blood oxygen level-dependent (BOLD) signal; electroencephalography (EEG) or magnetoencephalography (MEG) captures electrophysiological processes in the brain cortex directly. Several fMRI studies of resting-state FC found PD- and DT-related alterations (see [4] for a review). Typically, FC studies stop at analyzing the connection strength; a deeper understanding of the brain network’s architecture could be obtained using graph theory, amongst others. Moreover, graph representation allows for the evaluation of small-worldliness of the network, which is characterized by high integration and segregation [5]. Accordingly, an EEG study found higher segregation (captured in the normalized clustering coefficient) and lower integration (captured in the inverse of normalized path length) in the examined functional brain networks of PD patients during DT [6]. Although this study did not explore the brain dynamics of PD patients after an appropriate washout period of DT, a valid question could be whether the observed differences in the cortical networks could be attributed to the disease or to the pharmacological intervention. An extensive review by Tahmasian and colleagues of fMRI studies suggests that abnormalities in FC can emerge due to both PD and DT [7], while an EEG study showed increasing FC after DT [8]. These findings corroborate the use of brain network measures in the characterization of PD and DT; nevertheless, their interpretation would largely depend on the neuroimaging method, data processing steps and choice of different FC estimators.

Several different FC estimators can be used to study clinical populations (see [9] for an example in Alzheimer’s disease patients). These measures are typically tied to a specific time scale; on the other hand, studies investigating the scale-free (i.e., fractal) nature of FC [10] in PD are still missing. Scale-free dynamics refer to processes whose spectral power (P) inversely relates to the frequency (f) as: $P=f^{-\beta }$ [11, 12] in the univariate case, where β is a scale-free exponent termed as spectral index. By the same token, power–law relationships hold between f and cross-spectral power in the bivariate case. These correspond to the power-law decay of the autocorrelation [11,13] or cross-correlation [14] function in the temporal domain that indicate long-term memory, a characteristic feature of scale-free dynamics. Scale-independent investigation is necessary to quantify this ubiquitous property of physiological systems [11,15], and to yield estimators of FC for coupled processes Previously, we demonstrated that EEG captures multifractal coupled dynamics in the brain [16–18]. In these studies, we showed that such multifractal coupling of the brain is region- and state-dependent, warranting the use of bivariate multifractality measures for the investigation of pathological states, such as PD.

The objective of this study was to explore the PD- and DT-related alterations of functional brain networks defined by scale-free coupled dynamics between distinct cortical regions. We estimated multifractal FC — using bivariate focus-based multifractal (BFMF) analysis [16,17]—of eyes open resting-state EEG recordings of healthy volunteers and PD patients both on and off DT. We explored how PD and DT affect the multifractal FC of the brain cortex, where we found increased connectivity and integration along with decreased segregation of the networks after DT. We also investigated the organization of healthy cortical networks and how it relates with cognitive ability, suggesting that network metrics correlate with metrics of verbal intelligence.

## 2. Materials and Methods

### 2.1. Data Acquisition

We analyzed a publicly available (https://openneuro.org/datasets/ds002778/versions/1.0.3, accessed on 28 November 2020) EEG dataset consisting of recordings from 15 Parkinson’s disease patients (8 females, aged 62.6±8.3 years) and 16 community-dwelling elderly adults (HC) (9 females, aged 63.5±9.6 years) [19–23]. The PD patients were responsive under DT. HC was matched on age, handedness, sex, Mini-Mental Status Exam (MMSE) and North American Adult Reading Test (NAART) scores. EEG recordings of the PD patients were acquired during DT (PD-ON) and after at least 12 h without DT (PD-OFF), while HC’s EEG was recorded only once. United Parkinson’s Disease Rating Scale (UPDRS III) scores for PD were also assessed while ON and OFF medication. The EEG measurements lasted 3 min, with the participants being in a resting state with eyes open. The EEG recording device ActiveTwo (Biosemi Instrumentation System, The Netherlands) had 32 channels (according to the 10–10 system) and a sampling rate of 512 Hz. All participants provided informed consent based on the guidelines of an Institutional Review Board Protocol at the University of California, San Diego, USA. Details about the participants can be found in Tables 1 and 2 of the original publication [19].

### 2.2. Preprocessing

After visual inspection of the EEG recordings, eight continuous seconds of artifact-free signal were selected per participant for analysis. We transformed these epochs to reference-free current source density (CSD) time series using the spherical spline algorithm [24], as implemented in the CSD toolbox for MATLAB [25,26]; thus, spurious interdependencies due to volume conduction were minimized. Then, we band-pass filtered the EEG signals with lower and upper cutoff frequencies at 0.5 and 45 Hz, respectively, using the default finite impulse response filter in the EEGLAB toolbox for MATLAB [27]. The lower cutoff of 0.5 Hz was selected to match the lower range of δ EEG band, while the upper cutoff of 45 Hz was selected to avoid line noise at 50 Hz. Finally, we performed independent component analysis [28] using EEGLAB’s built-in functions and manually removed non-neuronal components from the signals.

### 2.3. Bivariate Focus-Based Multifractal Analysis

The multifractal nature of a connection can be described by its scaling exponents. These exponents represent the long-term cross-correlation of the connection, with different exponents being unequally influenced by large and small fluctuations. Here, we use two endpoints from BFMF analysis (see [16,17] for a more detailed explanation), characterizing the global long-term cross-correlation and the degree of multifractality. To obtain such measures, the scaling function of the coupled process should be constructed first.

**Figure 1.**
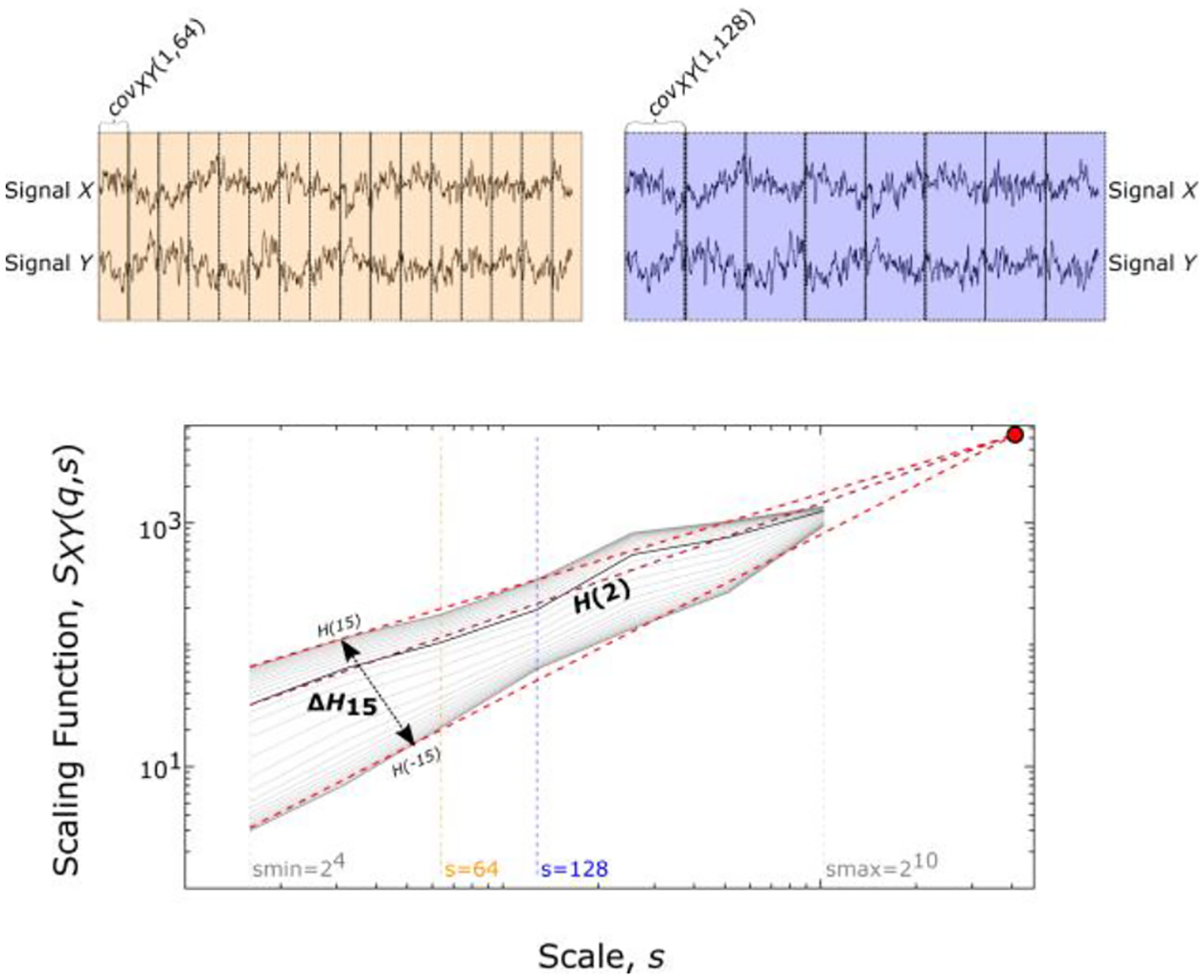
Endpoint parameters of multifractal time series analysis. The Focus (red dot) is used as a point of reference for simultaneously fitting linear models across data in the log-log transform of the scaling function $\left(S_{XY}\left(q,s\right)\right)$ vs. scale (s). The coefficient of each linear regression represents the generalized Hurst exponent H(q) at any given statistical moment q.H(2) describes the long-term cross-correlation between the signals X and Y, while the degree of multifractality $\left(\Delta H_{15}\right)$ is captured by the difference between H(q) at the minimal (q=−15) and maximal (q=15) statistical moments. The yellow and purple colors represent examples of scaling function estimation for s=64 and s=128, respectively. This is a modified version of the figure that first appeared in [18].

The scaling function, $S_{XY}$, of the two time series X and Y, in which the means have been subtracted as a centering step of length L datapoints is defined as follows:

(1) $$S_{XY}\left(q,s\right)={\left(\frac{1}{N_{s}}\overset{N_{s}}{\underset{v=1}{\sum }}{\left|{cov}_{XY}\left(v,s\right)\right|}^{q/2}\right)}^{1/q}$$

where q represents the statistical moment order, $N_{s}$ the number of non-overlapping windows of size s indexed by v and ${cov}_{XY}\left(v,s\right)$ the covariance of signals X and Y in window *v*. Each window is bridge-detrended before the calculation of ${cov}_{XY}\left(v,s\right)$. To reliably capture multifractality, q was set to range from −15 to 15 with increments of 1, as this selection of moment orders proved sufficient [29]. At the special case of q=0 the scaling function takes the form: ${\text{S}}_{\text{XY}}\left(0,\text{s}\right)={\text{e}}^{\frac{1}{2{\text{N}}_{\text{s}}}}\sum_{\text{v}=1}^{{\text{N}}_{\text{s}}}\text{log}\left(\left|{\text{cov}}_{\text{XY}}\left(\text{v},\text{s}\right)\right|\right)$. Since small and large time scales must be excluded due to statistical instability (too few datapoints or too few time windows for fractal analysis), s takes values of $2^{n}$ with n ranging from 4 to 10. Subsequently, linear models between log[s] and log[S(q,s)] for every q are fitted simultaneously using the so-called “*Focus*” as a reference point of the regression (Figure 1). Focus represents the point of convergence for all q as the scale approaches the signal length L. The slopes of this family of linear models yield the generalized Hurst exponent (H(q)):

(2) $$S\left(q,s\right)\propto s^{H\left(q\right)}$$

The end point parameters of the analysis are H(2) and $\Delta H_{15}$. H(2) corresponds to the generalized Hurst exponent for q=2 and indicates the global long-term interdependence between X and Y(H(2)=0.5 uncorrelated dynamics with H(2)<0.5 being short-term and H(2)>0.5 long-term cross-correlation) [10,11]. As shown in Equation (1) q are used as weights for the covariance of each segment. A large $\left|{cov}_{XY}\left(v,s\right)\right|$ is weighted more when q>0, while a small $\left|{cov}_{XY}\left(v,s\right)\right|$ is weighted more when q<0. When q=2 this weight is canceled out—since Equation (1) reduces to: $S_{XY}\left(q,s\right)={\left(\frac{1}{N_{s}}\sum_{v=1}^{N_{s}}\left|cov_{XY}\left(v,s\right)\right|\right)}^{1/2}$–reverting to the equivalent of detrended cross-correlation analysis [10] from which the long-term interdependence of X and Y can be estimated. The difference between H(−15) and H(15), termed $\Delta H_{15}$ indicates the strength of bivariate multifractality [29,30] and it is calculated according to:

(3) $$\Delta H_{15}=H\left(-15\right)-H\left(15\right)$$

As explained in the appendix of Ashkenazy and colleagues [31], linear coupling depends only on the cross-spectral power of the two signals comprising the connection; meaning that the cross-spectral slope—and by extension H(2) [12]—is a sufficient indicator of such interconnectivity. On the other hand, H(2) cannot capture non-linear dynamics that can be investigated by studying the other endpoint of our analysis, the width of multifractal spectrum (or degree of multifractality) using $\Delta H_{15}$.

### 2.4. Assessing Multifractality

To assess the true multifractal nature of the functional connections, the same array of tests as in Stylianou et al. [18] was utilized. A detailed description of the multifractality assessment tests can be found in the aforementioned publication; here, a summary is provided. The first test investigated the presence of a power–law relationship between frequency and cross-spectral power [12] of every pair of simultaneously recorded process. An extension of the detrended cross-correlation coefficient test proposed by Podobnik et al. [32] evaluated the scale-free character of the cross-correlations in the time domain as well. Next, the non-linear nature of the connection was tested by comparing the original $\Delta H_{15}$ to a distribution of $\Delta H_{15}$ obtained from phase-randomized surrogates [33]. Subsequently, H(2) and $\Delta H_{15}$ of the original functional connections were contrasted with those of shuffled datasets The purpose of this shuffling test was to investigate if the observed multifractality was due to long-term cross-correlations or resulted from spurious multifractality [34]. The final test determined the origin of multifractality by comparing the bivariate H(2) to the mean of its corresponding univariate Hurst exponents to classify the observed multifractality as either intrinsic or extrinsic to the connection. Whereas intrinsic bivariate multifractality represents scale-free coupling due to true functional interdependence [35], extrinsic multifractality can emerge from (i) the autocorrelation of the signals creating the connection [36] (ii) the finite length of the time series [37] or (iii) the non-normal distribution of the signals [38].

### 2.5. Brain Networks

Using the brain regions sampled by EEG as nodes and the connection strength between them as edges, we constructed functional brain networks revealing the topological organization of scale-free coupling. For each edge, we assigned a value quantifying the scale-free functional coupling as captured either by H(2) or $\Delta H_{15}$ for every dataset (i) HC:16H(2) and $16\Delta H_{15}$ networks, (ii) PD-ON: 15H(2) and $15\Delta H_{15}$ networks and (iii) PD-OFF: 15H(2) and $15\Delta H_{15}$ networks. We then calculated the weighted node degree $\left(D^{W}\right)$, weighted clustering coefficient $\left(C^{W}\right)$ and weighted path length $\left(L^{W}\right)$ and their averages across the whole graph (network): $\bar{D^{W}}$, $\bar{C^{W}}$ and $\bar{L^{W}}$. $D^{W}$ represents the strength of the scale-free coupled dynamics in a brain region, $C^{W}$ shows the degree of segregation and $L^{W}$ indicates how well an area is integrated in the network. The brain connectivity toolbox [39] was used to estimate these network metrics. Further details about the calculated network measures can be found in Rubinov et al. [39].

### 2.6. Statistical Evaluation

Firstly, the global network properties ($\bar{D^{W}}$, $\bar{C^{W}}$ and $\bar{L^{W}}$) were contrasted between the three different groups (HC, PD-ON and PD-OFF). For the HC vs. PD-OFF and HC vs. PD-ON comparisons, we used non-paired tests, since we had different participants in every group. The normality of each distribution was assessed using the Lilliefors test. Lilliefors test compares the distance between the original distribution and a hypothesized normal distribution with mean and variance based on the sample. If the maximal distance exceeds the threshold necessary for being statistically significant the distribution in question is not normal. If the normality assumption was violated by at least one of the distributions, we proceeded with the Mann-Whitney U-test. If both distributions were normal, we performed a two-sample F-test to investigate the relationship between the variance of the two distributions. If the variances were statistically similar (p>0.05), we continued with the two-sample t-test; while otherwise, the Welch’s t-test was used. In the case of the PD-ON vs. PD-OFF comparisons, we used paired tests, since the same participants were studied ON and OFF medication. These tests were the Wilcoxon signed rank test or paired t-test, depending on the normality of the distributions (assessed by the Lilliefors test). The same statistical pipeline was carried out for the local network properties ($D^{W}$, $C^{W}$ and $L^{W}$) comparisons, which were then adjusted using the Benjamini–Hochberg (BH) correction [40].

An important prerequisite of subsequent analysis was the validation of the effect of DT. We compared the UPDRS III of PD as collected in their two visits (ON and OFF medication) using a paired-sample t-test, since both distributions were normal (assessed by the Lilliefors test). Finally, we studied the relationship between the different score results (MMSE and NAART) and the global network properties ($\bar{D^{W}}$, $\bar{C^{W}}$ and $\bar{L^{W}}$) in HC. Firstly, we assessed the normality of the distributions using Lilliefors test. If both distributions were normal, we calculated the Pearson’s correlation between them. Since Pearson’s correlation is not suitable for non-normal distributions, if at least one distribution was not normal we calculated Spearman’s correlation. Subsequently, the p values were adjusted using the BH procedure. Since it was not known which PD patients took the MMSE and NAART tests ON or OFF medication, we could not do any correlation analysis for the PD-OFF and PD-ON groups.

## 3. Results

Table 1 summarizes the percentage of connections that passed each of our multifractality assessment tests. The highest success rate was found in the phase randomization and shuffling tests, followed by the spectral slope test. On the other hand, less than a quarter of the connections were found to have intrinsic multifractality (investigated with bivariate-univariate comparison test) or power-law dynamics in the time domain (detrended cross-correlation test). We also see in Table 2 that less than 1% of the connections passed all the tests.

**Figure 2.**
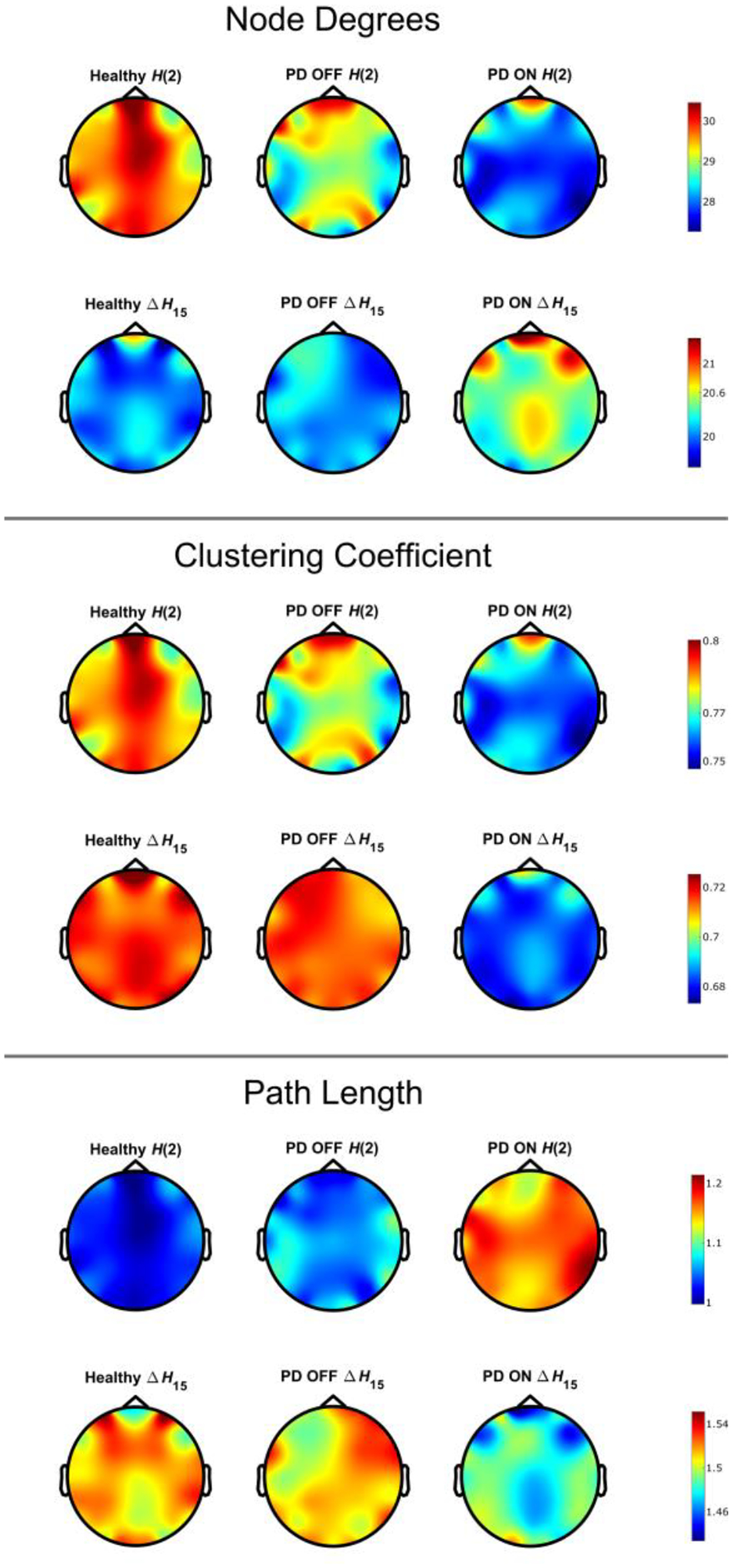
Distribution of the network metrics in the cortex. HC: healthy control, PD OFF: Parkinson’s disease patients off medication, PD ON: Parkinson’s disease patients on medication.

**Figure 3.**
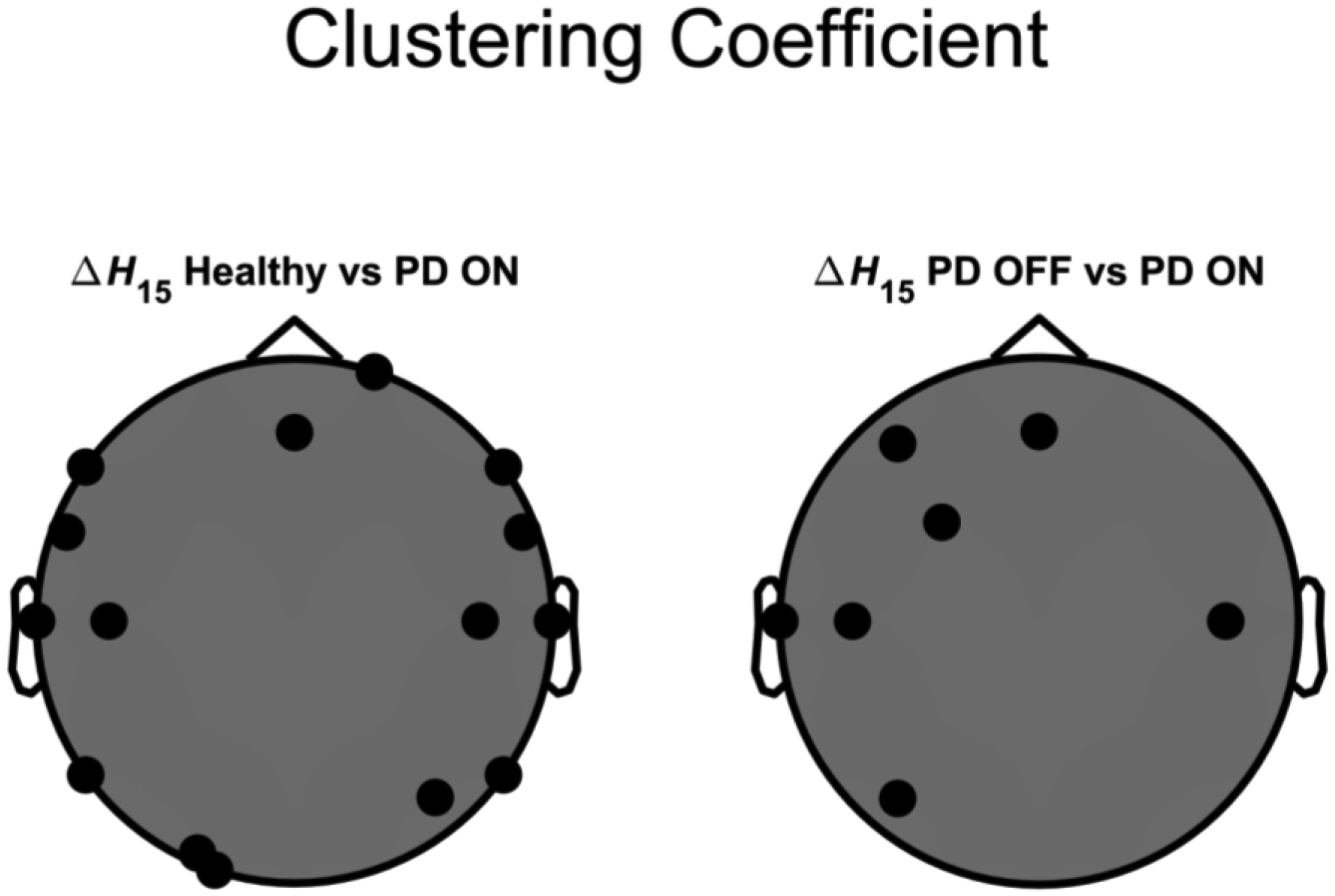
Brain regions with significantly different clustering coefficients in ${\Delta \text{H}}_{15}$ networks. The black disks represent the regions whose comparison was statistically significant, i.e., Benjamini–Hochberg adjusted p-value < 0.05. HC: healthy control, PD OFF: Parkinson’s disease patients off medication, PD ON: Parkinson’s disease patients on medication.

We then proceeded with the construction and comparison of the BFMF-derived networks. As a first step, we compared the global network properties of the three different groups. As can be seen in Table 3, the PD-ON state was significantly different (BH-adjusted p-value <0.05) from PD-OFF and HC in every network measure ($\bar{D^{W}}$, $\bar{C^{W}}$ and $\bar{L^{W}}$) for the $\Delta H_{15}$ networks, but not for H(2) networks. Specifically, we found increased $\bar{D^{W}}$ along with decreased $\bar{C^{W}}$ and $\bar{L^{W}}$ in PD-ON, compared to HC and PD-OFF groups for the $\Delta H_{15}$ networks (Figure 2). Regarding the local comparisons, significant differences (BH adjusted p-value <0.05) were only found in the $C^{W}$ between PD-ON and HC (channels: FC5, P8, C4, FC6, F8, Fz, F7, T7, C3, P7, PO3, O1, P4, T8 and Fp2) and between PD-ON and PD-OFF (F3, FC1, T7, C3, P3, C4 and Fz) in the $\Delta H_{15}$ networks (Figure 3). Additional figures about the local comparisons can be found in the Supplementary Material (Figure S3).

**Figure 4.**
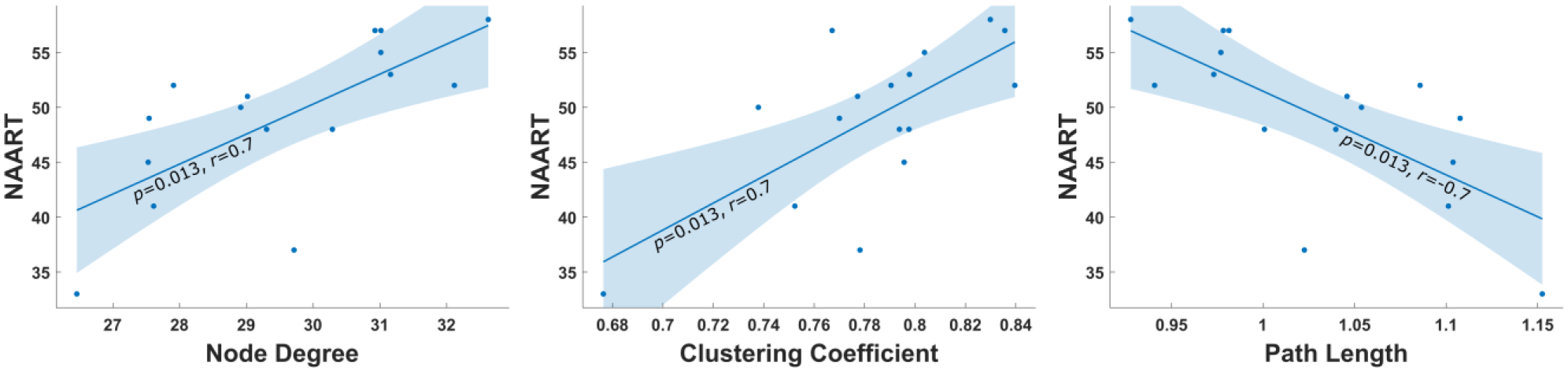
Linear regressions of H(2) network metrics and NAART scores. The indicated p-values are adjusted based on Benjamini-Hochberg correction.

The comparison of the UPDRS III scores between the PD-ON and PD-OFF groups showed that during DT the UPDRS III scores decreased (p=0.001). We also found significant correlations (BH adjusted p-value <0.05) between the global metrics of H(2) networks and the NAART scores in the HC group. $\bar{D^{W}}$ and $\bar{C^{W}}$ were positively correlated (r=0.7), while $\bar{L^{W}}$ was negatively correlated (r=−0.7) (Figure 4). No correlations were seen between network metrics and MMSE scores.

## 4. Discussion

In the present study, we characterized multifractal coupled dynamics of healthy and PD populations as they were captured in EEG tracings. Pharmacological administration significantly improved the motor symptoms of the disease, as seen in the UPDRS III scores. The architecture of the $\Delta H_{15}$ networks changed due to DT, specifically the interconnectivity and integration increased, while the segregation of the network decreased. Finally, NAART scores correlated with the architecture of the H(2) networks of control participants.

As seen in Table 1, several connections passed the multifractality assessment tests, similar to our previous studies [17,18]. Yet, only a small fraction of them passed all the tests (Table 2). This is in disagreement with our previous findings in Stylianou et al. [18], where more than 50% of the connections passed all tests during eyes open resting state. The HC population in the current study was much older than the analyzed population in Stylianou et al. It is possible that the multifractal profile of FC diminishes with age, which has not been studied yet. The network dynamics, as captured by the long-term cross-correlation of the connections (H(2)), did not differ between the three groups, but we found significant differences in the degree of multifractality of the connections $\left(\Delta H_{15}\right)$ (Table 3, Figure 2). The two BFMF outputs capture complementary aspects of scale-free dynamics, explaining why different network architectures might emerge [17]. Additionally, H(2) is an estimator of linear coupling, while $\Delta H_{15}$ depends on non-linear dynamics. We can then conclude that the linear relationship between regions was not different, while non-linear dynamics—captured by $\Delta H_{15}$ — were altered after DT.

Three major dopaminergic pathways reach the cortex, each one associated with different symptoms of PD: (i) nigrostriatal, (ii) mesocortical and (iii) mesolimbic [41]; the tuberoinfundibular pathway does not reach the cortex. The mesolimbic pathway projects to the cingulate cortex, whose activity cannot be directly inferred from scalp EEG. The influence of the nigrostriatal and mesocortical pathways can be evaluated more directly by EEG, since these pathways project to the motor and prefrontal cortex, respectively. The prefrontal cortex is a high-level association area with abundant incoming and outgoing connections, meaning that changes in the mesocortical pathway would have a greater influence on the cortical EEG, compared to alterations of the mesostriatal pathway. In the meantime, the mental fitness of the HC was matched to that of the PD population, based on their MMSE and NAART scores. This suggests that the prefrontal cortex of PD was intact relative to HC, consistent with the lack of significant differences between HC and PD-OFF. On the other hand, the administration of DT affected the mesocortical circuitry and, by extension, a large portion of the cortex, resulting in differences between HC and PD-ON, as well as PD-OFF and PD-ON. Administering DT directly means that brain regions with dopamine receptors that are not part of the dopaminergic pathways could also be affected. Nonetheless, dopamine receptors are mainly concentrated in regions that are part of the aforementioned circuitries [42,43], meaning that their involvement is the most probable reason for the found alterations.

Compared to FC estimation at the level of individual connections, graph theoretical parameters provide further insights into the architecture of the functional brain networks. Hence, we calculated network metrics that describe the interconnectivity $\left(D^{W}\right)$, segregation $\left(C^{W}\right)$ and integration $\left(L^{W}\right)$ of the networks. $\bar{D^{W}}$ was elevated after DT in the $\Delta H_{15}$ networks (Table 3, Figure 2), meaning that the treatment itself increased the degree of multifractality (an indicator of non-linear coupling) of the connections between the different cortical regions. A similar rise of non-linear coupling in treated PD patients—albeit using another FC estimator (synchronization likelihood)—was found in a previous MEG study [44]. Another EEG study showed both increase and decrease in node degree using a non-linear FC estimator (phase lag index or PLI) in treated PD patients; node degree increased in the theta and beta bands of PD patients, while it decreased in the delta and alpha bands [6]. We also found a decrease in $\bar{C^{W}}$ and $\bar{L^{W}}$ after DT. Utianski et al.—using PLI—showed that $\bar{C^{W}}$ and $\bar{L^{W}}$ increased in treated PD patients, compared to matched healthy controls [6]. On the other hand, Dubbelink et al.-also using PLI-in a MEG study found that $\bar{C^{W}}$ and $\bar{L^{W}}$ decreased in PD patients during DT, compared to healthy controls [45]. In Dubbelink et al., even though these changes are not explicitly stated, they can be inferred from Figure 3A. A major difference between our work and the studies of Utianski et al. and Dubbelink et al. is that we constructed brain networks based on the BFMF outputs, while they used PLI, a non-linear but scale-specific estimator of FC. We then decided to re-analyze our dataset but this time using PLI as an FC estimator. The results showed that both $C^{W}$ and $\bar{C^{W}}$ were significantly lower in the PD-ON, compared to HC, i.e., agreeing with Dubbelink et al However, local and global measures of interconnectivity $(D^{W}$ and $\bar{D^{W}})$ or integration $(L^{W}$ and $\bar{L^{W}}$) did not show PD-related differences. Details about this analysis can be found in the Supplementary Material. It has been demonstrated that functional brain networks show small-world properties [46,47], meaning that such networks are characterized by high segregation and integration [39]. The decrease in $\bar{C^{W}}$ and $\bar{L^{W}}$ of BFMF networks indicates lower segregation and higher integration during DT. No differences were seen in the OFF state compared to HC, which was only a few hours apart from the ON state, suggesting that the observed alterations of small-world character are not due to pathological changes of PD but rather caused by pharmacological influences. It is of note that DT did not exclusively heighten or dampen the small-world properties of the network. Similar inconsistencies of small-worldliness were seen in Vecchio et al. [48], where the small-world index (ratio of normalized clustering coefficient and normalized path length) was higher in PD in the alpha 2 but lower in the theta EEG band. Such complex interactions suggest that we should study the influence of DT in the small-world architecture of cortical networks even further. In terms of local network metrics, we found significant differences only in $C^{W}$ when we compared HC vs. PD-ON and PD-OFF vs. PD-ON. These differences were mainly localized in the periphery of the cortex (Figure 3). Prior to BH correction we had significant differences in $D^{W}$ and $L^{W}$ as well. We believe that a bigger sample size could show significant differences in $D^{W}$ and $L^{W}$, even after adjusting the p-values.

Another question we sought to answer was if such network metrics correlate with mental capacity, which could be used as clinical biomarkers. We did not find any significant correlations between the estimated network metrics and MMSE scores in HC. On the other hand, the metrics of the H(2) networks correlated with the NAART scores (Figure 4) [49]. Higher NAART scores associated with higher interconnectivity, segregation and integration of the brain networks, i.e., stronger small-world characteristics. NAART test is a reliable indicator of verbal intelligence [49]. This agrees with the notion that small-world networks are more efficient in information processing, which in this case was higher verbal intelligence. The lack of correlations between scale-free network metrics and MMSE due to the preliminary nature of those findings should not be regarded conclusive.

Despite the novelty of our findings, the following limitations should be outlined. MMSE and NAART scores of the PD patients were provided without indicating which patients took the tests ON and which took the tests OFF medication. This did not allow us to investigate the possible correlations of the scores with the network metrics in the PD population. Additionally, because of the small sample size, we cannot make inferences of the population. This is a common problem in studies where clinical populations are recruited. A possible solution to this issue is the generation of simulated EEG signals [50,51] based on structural connectivity or FC obtained through other imaging modalities, a methodology already implemented in an Alzheimer’s disease study [52]. Finally, in the last decade there have been growing indications that crucial events can generate multifractality [53,54]. It would be then beneficial to explore such theories in clinical populations, such as PD patients.

## 5. Conclusions

In this study, we investigated the multifractal functional connectivity of healthy and Parkinson’s disease participants. The results showed that the degree of multifractality was altered during dopaminergic treatment. By using a series of metrics, we showed that the complex architecture of the cortical network exhibited increased interconnectivity and integration and decreased segregation. We postulate that this difference can be attributed to the pharmacological administration of dopamine exerting a widespread short-term effect on functional cortical networks, since EEG could not detect differences between healthy control and PD patients when they were off medication. Finally, the verbal intelligence of the healthy participants depended on the small-world architecture of the network, paving the way for deeper investigation of small-world properties in scale-free FC networks.

## Supplementary Material

supplemental material

## Figures and Tables

**Table 1.** Success rate of multifractality tests at the subject level (mean ± standard deviation).

Group	Tests					
	SS	PR	$\text{S}\Delta H_{15}$	S-H(2)	DCCC	Biv-Univ
HC	78 ± 4%	97 ± 4%	100 ± 0%	100 ± 0%	11 ± 7%	10 ± 5%
PD-OFF	77 ± 4%	99 ± 1%	100 ± 0%	100 ± 0%	8 ± 4%	11 ± 7%
PD-ON	72 ± 15%	99 ± 2%	100 ± 0%	94 ± 14%	11 ± 9%	20 ± 19%

SS: spectral slope test, PR: phase randomization test, $\text{S-}\Delta H_{15}:\Delta H_{15}$ part of the shuffling test, S-H(2):H(2) part of the shuffling test, DCCC: detrended cross-correlation coefficient test, Biv-Univ: bivariate-univariate H(2) comparison, HC: healthy control, PD-OFF: Parkinson’s disease patients off medication, PD-ON: Parkinson’s disease patients on medication.

**Table 2.** Percentage of connections at the subject level (mean ± standard deviation) that passed all multifractality assessment tests.

Network	Group		
	HC	PD-OFF	PD-ON
H(2)	0.7 ± 1%	0.4 ± 0.4%	0.8 ± 0.7%
$\Delta H_{15}$	0.7 ± 0.8%	0.4 ± 0.4%	0.8 ± 0.7 %

HC: healthy control, PD-OFF: Parkinson’s disease patients off medication, PD-ON: Parkinson’s disease patients on medication.

**Table 3.** Benjamini–Hochberg adjusted p-values.

	$H\left(2\right)\;\bar{D^{W}}$	$\Delta H_{15}\;\bar{D^{W}}$	$H\left(2\right)\;\bar{C^{W}}$	$\Delta H_{15}\;\bar{C^{W}}$	$H\left(2\right)\;\bar{L^{W}}$	$\Delta H_{15}\;\bar{L^{W}}$
HC vs. PD-OFF	0.43	0.62	0.48	0.76	0.34	0.62
HC vs. PD-ON	0.86	0.03*	0.26	0.01*	0.83	0.03*
PD-OFF vs. PD-ON	0.19	0.04*	0.85	0.02*	0.19	0.04*

HC: healthy control, PD-OFF: Parkinson’s disease patients off medication, PD-ON: Parkinson’s disease patients on medication, $\bar{D^{W}}$: global node degree, $\bar{C^{W}}$: global clustering coefficient, $\bar{L^{W}}$: global path length,

*: p values smaller than 0.05.

## Data Availability

https://openneuro.org/datasets/ds002778/versions/1.0.3.
