# Supplementary material for: Scale-Free Functional Brain Networks Exhibit Increased Connectivity, Are More Integrated and Less Segregated in Patients with Parkinson’s Disease following Dopaminergic Treatment
Source: Fractal Fract. Author manuscript; Available in PMC 2023 Dec 15. (PMC10723163; doi:10.3390/fractalfract6120737)
Supplement: supplemental material [file NIHMS1904669-supplement-supplemental_material.pdf]

Phase-Lag Index Analysis

In order to compare our analysis with the results of Dubbelink et al. (Olde Dubbelink et al., 2014) we estimated the brain's functional connectivity (FC) using phase-lag index (PLI) (Stam et al., 2007). After extracting the FC matrix for every subject, we calculated the same network metrics as in the main analytical pipeline (i.e. global/local node degree, global/local clustering coefficient and global/local path length).

Once again, we compared the network metrics between the three groups (HC, PD-ON and PD-OFF) in a series of paired comparisons. The normality of each distribution was assessed using the Lilliefors test. If the normality assumption was violated by at least one of the distributions, we proceeded with the Mann-Whitney U-test in case of HC vs PD-OFF or HC vs PD-ON comparison. If both distributions were normally distributed, we performed a two-sample F-test to investigate the relationship between the variance of the two distributions. If the variances were statistically similar ($p>0.05$), we continued with the two-sample t-test; while otherwise, the Welch's t-test was used. For the PD-ON vs PD-OFF comparisons, we used the Wilcoxon sign rank test or paired t-test, depending on the normality of the distributions (assessed by the Lilliefors test). The same statistical pipeline was carried out for the local network properties comparisons which were then adjusted using the Benjamini-Hochberg (BH) correction (Benjamini and Hochberg, 1995). As seen in **Figure S1**, statistically significant differences (BH adjusted *p*-value <0.05) were found only in the comparison between Healthy and PD-ON, where DT decreased the global clustering coefficient. The same pattern is seen (**Figure S2**) in the local network metrics comparisons where only the local clustering coefficient was significantly different between HC and PD-ON (channels: P7, AF3, PO4, CP6, CP2, FC1, O1, P8, T8, Fz, Cz).

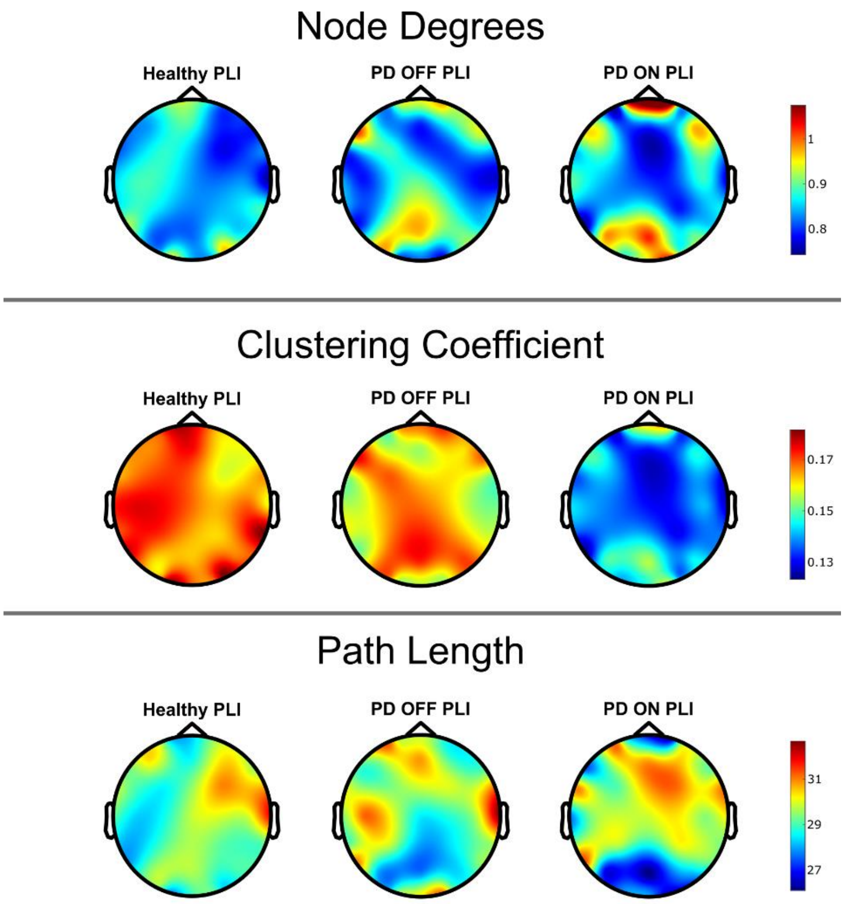

Figure S1. *Distribution of the network metrics in the brain cortex.* HC: Healthy control, PD OFF: Parkinson's disease patients off medication, PD ON: Parkinson's disease patients on medication

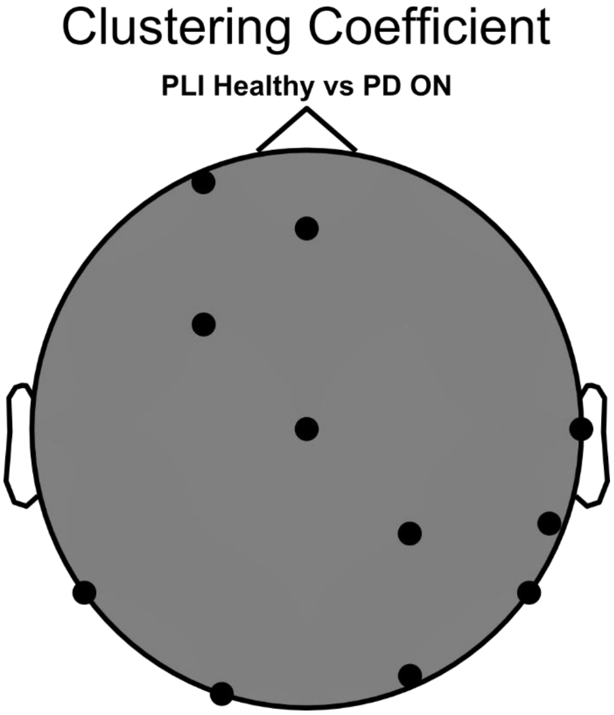

Figure S2. *Brain regions with significantly different clustering coefficients in PLI networks.* The black disks represent the regions whose comparison was statistically significant, i.e. Benjamini-Hochberg adjusted *p*-value <0.05. HC: Healthy control, PD ON: Parkinson's disease patients on medication

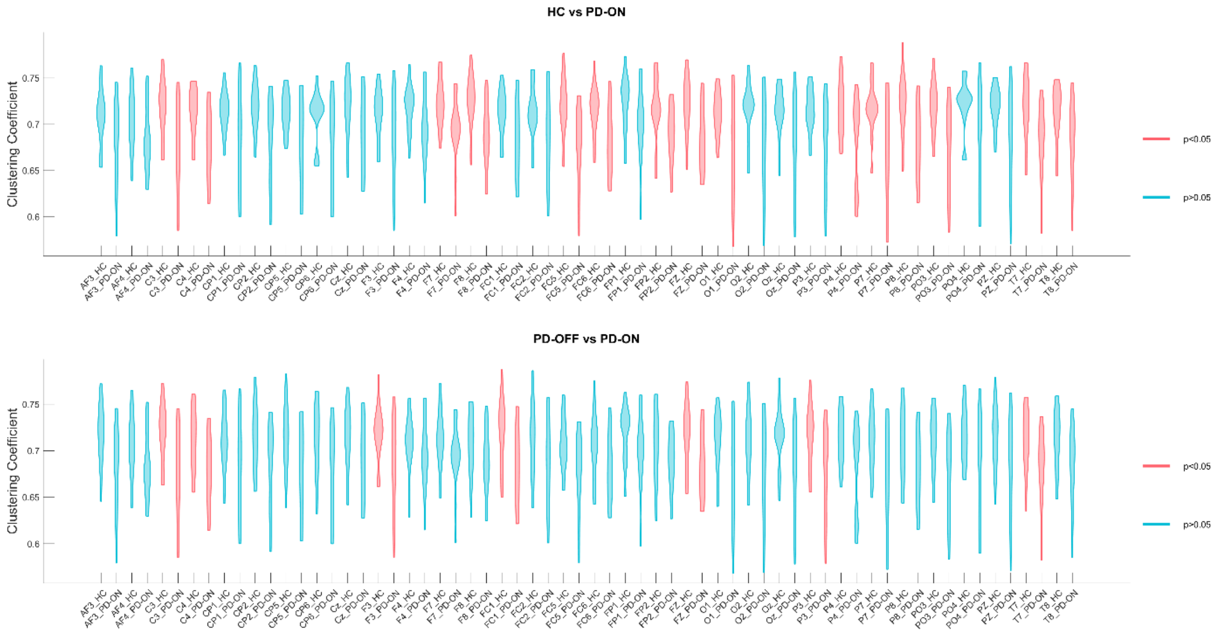

Figure S3. *Violin plots of the local clustering coefficients of the ΔH_{15} networks.* The red distributions indicate the comparisons whose Benjamini-Hochberg adjusted *p*-value was smaller than 0.05. HC: Healthy control, PD OFF: Parkinson's disease patients off medication, PD ON: Parkinson's disease patients on medication
